# Congenital hypothyroidism and associated visual-motor and intellectual development

**DOI:** 10.1038/s41390-025-03850-3

**Published:** 2025-01-22

**Authors:** Esperanza Ontiveros-Mendoza, Juan Antonio González-Medrano, Rolando Rivera-González, Karla Sánchez-Huerta, Carmen Sánchez, Gerardo Barragán-Mejía

**Affiliations:** 1 Centro de Investigación en Neurodesarrollo. Instituto Nacional de Pediatría, Mexico City, México; 2 Universidad Autónoma Metropolitana-Unidad Xochimilco, Mexico City, México; 3 Laboratorio de Neurociencias. Instituto Nacional de Pediatría, Mexico City, México; 4 Laboratorio de Bacteriología Experimental. Instituto Nacional de Pediatría, Mexico City, México

## Abstract

**Background:**

Congenital hypothyroidism’s sequelae include visuomotor and intellectual developmental deficits. Visual-motor perception is a cognitive function related to academic performance. Intellect is the ability to learn and use acquired knowledge to solve and achieve goals. Our objective was to evaluate visual-motor and intellectual development in children with late initiation of treatment for congenital hypothyroidism enrolled in a developmental follow-up and intervention program.

**Methods:**

We evaluated the visual-motor and intellectual development of 75 infants with congenital hypothyroidism, 34 with athyrosis, and 41 with ectopia using the Bender Visual-Motor Development Test and the Weschler Intelligence Scale at eight and nine years of age.

**Results:**

Children with ectopia had a visual-motor delay of −2 years and an Intelligence Quotient (IQ) greater than 98 points. Children with athyrosis had a visual-motor delay equivalent to −3.2 years and an IQ below 90 points. Better performance on Bender’s test was positively correlated with IQ. Attending more than 80% of Developmental Intervention Program appointments had a positive impact on intellectual development.

**Conclusions:**

Timely diagnosis and early treatment with the appropriate dose of levothyroxine are determining factors; however, attendance to a development follow-up and intervention program could further support the cognitive development of children with congenital hypothyroidism.

**Impact:**

- Visuomotor development influences cognitive functions related to school performance.
- Deficits in the ability to learn and use acquired knowledge to solve problems and achieve goals are characteristic of children with congenital hypothyroidism.
- Delayed treatment is associated with more severe sequelae in both visual-motor and intellectual development.
- Here, we show that treatment with adequate doses of levothyroxine and inclusion in a follow-up and developmental program are highly recommended for patients with delayed treatment.
- A better understanding of these factors will allow clinicians to target therapeutic interventions.

## Introduction

Congenital hypothyroidism (CH) is a disease characterized by the deficiency of thyroid hormones at birth. The most frequent cause of primary CH is an abnormal development of the thyroid gland or dysgenesis (80-90%) and its most common form of presentation is ectopia (65%), followed by athyrosis (absence of the thyroid gland) which occurs in approximately 30% of cases. The global prevalence of CH is 1 in every 2000 to 3000 live newborns.^1^ In Mexico, Vela-Amieva et al. ^2^ reported a prevalence of 4.12 cases per 10,000 newborns, and for the year 2018, it was 7.3 per 10,000 newborns,^3^ with a female-to-male ratio of 2:1, indicating a predominance among females.^4^

Neonatal screening is a fundamental tool for timely detection, early initiation of treatment, and limiting the neuropsychological sequelae of CH. Coverage of neonatal screening has increased steadily, reaching up to 96% in some European countries.^5^ However, in Latin America, coverage is still below the ideal, which implies a higher risk of neuropsychological sequelae in the undiagnosed and lately treated population.^6^

CH is associated with neuropsychological deficits that have been identified in individuals from the earliest years of life to early adulthood. It affects a number of neurocognitive domains including intelligence quotient (IQ), language, learning, memory, and sensorimotor, visuospatial, and visuoconstructive abilities.^7^ Specifically, studies have demonstrated that children with CH exhibit lower global, verbal, and performance IQ scores compared to their siblings or healthy children.^8–17^ Additionally, alterations in motor and visual functions have been observed. Children with CH display deficits in motor coordination and visual-motor precision, as well as prolonged reaction time,^18–20^ diminished performance on block design and object assembly,^21^ and a reduced capacity to detect and process visual stimuli.^22^

The evidence indicates that the timing of initiation of the treatment and the etiology are determining factors of the neuropsychological outcome in patients with CH.

According to the current recommendations, levothyroxine (L-T_4_) treatment (10–15 µg/kg/day) should be initiated no later than 2 weeks after birth or immediately after confirmation of serum test results,^1^ with the goal of maintaining TSH, free T_4_ or total T_4_ within the upper age-specific reference range.^23,24^ The evidence supports that as the timing of treatment initiation is delayed, IQ scores are lower. Thus, children with CH exhibited lower IQ scores when L-T_4_ treatment began after 29 or 30 days of age in comparison with CH children with early treatment.^25–27^ In contrast, CH children with optimum L-T_4_ treatment exhibit IQ scores within the reference values.^28–34^ With regard to the etiology of the CH, findings from multiple studies show that athyrosis represents the primary risk factor for significant deficits in IQ, language, sensorimotor and visuospatial functioning. Children with this condition exhibited lower full-scale, verbal and performance IQ compared to those with ectopia or dyshormonogenesis.^35–37^ Similarly, sensorimotor and visuospatial domains are also affected, children with CH due to athyrosis demonstrated deficient performance in eye-hand coordination and locomotion compared with children with CH due to ectopia or dyshormonogenesis.^35^

Visuo-perception is a process or cognitive function that integrates visuospatial skills, spatial structuring, the use and manipulation of objects (visuo-construction), visual analysis capacity (visual closure, constancy of form, and figure-background), fine and gross motor skills, and visual tracking and stimulus discrimination.^38^ It enables the processing of visually perceptible environmental stimuli, facilitating the identification and assimilation of various aspects of the environment. It is imperative to assess the impact of CH on visuo-perception, as visuomotor development is related to other functions, such as graphism and academic performance.

Children with severe CH who are treated late (with the onset of treatment greater than two weeks of age) achieve cognitive development that places them in unfavorable conditions with respect to those with mild CH or early L-T_4_ treatment.^11,39^

This raises the question of whether there are alternative treatments for these patients that can promote their development to levels that allow them to learn and take advantage of the acquired knowledge to solve problems and achieve goals at school age.

It can be surmised from prior experience that these alternatives have arisen from three distinct areas of study: the examination of different schemes of hormone replacement therapy, the investigation of neuropsychological sequelae of children with CH, and the assessment of the influence of integrating patients into intervention programs.

In this sense, there is evidence that intervention programs with a primary focus on neurodevelopment and the quality of interactions between parents and children have demonstrated to exert a beneficial influence on children’s cognitive development.^40–42^

Our hypothesis is that an initial treatment regimen with levothyroxine appropriate for the severity of CH and the incorporation of children into a developmental intervention and follow-up program are factors that can promote development in children even when treatment has been initiated at a late stage.

The objective of this study is twofold: firstly, to evaluate the visual-motor and intellectual development in school-age children with CH due to athyrosis or ectopia; and secondly, to identify whether the treatment regimen and incorporation into the follow-up program had any effect on their development.

## Methods

### Participants

This was a prospective study conducted with 75 infants, 56 females and 19 males, 34 with a confirmed clinical diagnosis of athyrosis (AT) and 41 with ectopy (EC) who were referred by the Endocrinology Department to the Neurodevelopment Research Center of the National Institute of Pediatrics.

The children were enrolled in a development follow-up and intervention program after the initial diagnosis, and their visual-motor and intellectual development was evaluated at 8 and 9 years with the Bender test and the Wechsler Intelligence scale.

### Endocrinological evaluation

Diagnosis of CH and treatment with L-T_4_ were carried out by endocrinologists in the Endocrinology department. Thyroid hormones were quantified following the standardized method used in the institution, and the etiology of CH was determined by a scintigraphy study with technetium 99.

### Neuropsychological evaluation

Neurodevelopment assessments were performed with two instruments, the Bender-Gestalt Test (Koppitz adaptation)^43^ and the Wechsler Intelligence Scale for School children (WISC-R),^44^ by highly qualified psychologists assigned to the Neurodevelopment Research Center. Each patient was evaluated in a single session lasting 60 to 90 minutes. The instruments were applied independently in a distraction-free environment, and a 15-minute break was allowed between each assessment.

The Bender test consists of nine cards (A, 1, 2, 3, 4, 5, 6, 7, and 8) with geometric patterns of varying complexity; the cards are presented individually and consecutively. The child is asked to copy the patterns, one by one, on a white letter-size sheet. When one copy is complete, the next card is presented until the nine are completed. Subsequently, the evaluator determines the presence (1 point) or absence (0 points) of distortions (errors) in each of the nine patterns drawn by the child. The distortions are classified into seven categories: distortion of shape or pattern disproportion, pattern rotation, replacing of dots for circles or lines, drawing perseverance, failure to integrate parts of a pattern, replacing curves for angles, and addition or omission of angles. Each of the distortions is assessed in the reproductions of the different patterns, with the presence of distortions scored as one point. The maximum number of distortions that can be recorded is 30; therefore, high scores indicate a lower level of visual-motor maturation. The data obtained can be analyzed in 3 ways: a comparison of the performance of the subject (number of errors) with the performance of other children of the same chronological age using normalized scores, a comparison of the performance of the subject with the performance of children in the same school grade or an assessment of the performance of the subject based on the level of visual-motor development that corresponds to his or her age.

In this study, the degree of visual-motor development expressed as visual-motor maturation age in years and months was determined, for which the score obtained in the Bender test was used to calculate the equivalent age referred to in the corresponding table of the test.^45,46^ For example, a score of 3 (3 errors in the reproduction of the patterns) equates to a maturation age of between 8 years 6 months and 8 years 11 months. From the calculation of the difference between the chronological age of each child and the maturation age (using the upper age limit of the table), an index was obtained as a measure of the degree of the development of visual-motor maturity. In addition to counting the number of errors, the most frequent types of errors found in the reproduction of the patterns by the children in the different study groups were determined.

IQ was evaluated using the Wechsler intelligence scale, in particular, the WISC-R. This test covers an age range from 6 years to 16 years 11 months, and three quotients are obtained: a verbal intelligence quotient (VIQ), a performance intelligence quotient (PIQ), and a global intelligence quotient (GIQ). The natural scores for each of the six subtests that comprise the WISC-R are added; the value is transformed into a normalized score and a quotient value, with scores ranging from 90 to 109 considered normal. The equivalent age of development was obtained from the ages indicated in the corresponding test tables for each of the scales: verbal, performance, and global. These data were used to determine the difference between chronological age and developmental age, from which an intellectual development index was obtained.

### Development follow-up and intervention program

Children enrolled in the program were periodically monitored for neurological, physical, and cognitive development, month by month in the first year, every two months in the second year, every three months in the third year, and then at an interval of 6 months. Early intervention, speech therapy and, where appropriate, cognitive therapy were provided. In addition, the children participated in playful and semi-academic activities, parents were given guidance on handling the child, and most importantly, they were encouraged to actively participate in the development of their own children. Trained personnel working at the Neurodevelopment Follow-up Center in the National Institute of Pediatrics participated in these activities: neurodevelopmental doctors, psychologists, speech therapists, various therapists, and social workers.

#### Data analysis

The objective of this study was to test the hypothesis that adequate pharmacological treatment and incorporation into a neurodevelopmental intervention and monitoring program could be beneficial for neurodevelopment in children with CH who initiated treatment at a late stage. To this end, data from children with CH due to athyrosis or ectopia at 8 and 9 years of age were analyzed.

In order to explore whether even, when treatment of children starts later than the existing recommendation, children with CH could reach a visual-motor and intellectual development within acceptable limits, a cut-off point was established to divide children into two groups: early (<30 days of age at the time of treatment initiation) or late (>30 days of age at the time of treatment initiation).

The cut-off value for dividing the children into two groups based on the initial dose of L-T_4_ was the intermediate value of the recommended initial dose (10–15 µg/kg/day) (1). Therefore, high doses of L-T_4_ were defined as >12.5 µg/kg/day, and low doses of L-T_4_ were defined as <12.5 µg/kg/day.

To determine whether regular attendance at the developmental intervention and follow-up program had any effect on these children’s development, a cut-off point of more than 80% attendance at intervention program appointments was used.

For the Statistical analysis continuous numerical variables are described by measures of central tendency and dispersion (mean, standard deviation). To compare differences in developmental variables by type of CH, age, initial hormonal levels, L-T_4_ dose, and exposure to the intervention program, analysis of variance (ANOVA) or Kruskal‒Wallis tests were used, followed by multiple comparisons, after confirming the existence of homogeneous variances. Tests were also used to measure the correlation between visual-motor variables and IQ. JMP v.11 software (SAS Institute) was used for the analyses.

## Results

### Age at initiation of treatment, initial thyroid profile, and L-T_4_ dose

The mean age at the initiation of treatment was 43 ± 18 days, 28 days longer than the recommendation established by the Congenital Hypothyroidism Consensus Guidelines Update 2020–2021 (1). The mean time to start treatment was similar for athyrosis or ectopia (~44 days) groups.

The serum concentration of free T_4_, thyroid stimulating hormone (TSH) and the initial L-T_4_ dose (at the time of diagnosis) were analyzed for the patients by type of CH. In the group of patients with athyrosis, the initial free T_4_ concentration was 0.39 ± 0.36 ng/dl, significantly lower (*p* = 0.002) than that for children with ectopia, 0.70 ± 0.51 ng/dl. For TSH concentration, although the values were slightly higher in the athyrosis group, there were no significant differences between the groups.

The average dose of L-T_4_ administered to all children was 13.6 ± 1.3 µg/kg/day; for children with ectopia, the dose of L-T_4_ was 13.4 ± 1.3 µg/kg/day, which is lower than that for the children in the athyrosis 13.9 µg/kg/day (Table 1).

**Table 1:** Mean age at treatment initiation, serum concentration of free T_4_ (f T_4_), thyroid stimulating hormone (TSH) and initial dose of L-T_4_.

	Treatment initiation	fT4 ng/dl	TSH mIU/ml	Doses L-T4 µg/kg/Day	*N*
**Athyrosis**	44 ± 21	0.39 ± 0.36**	75 ± 17	13.9 ± 1.3	34
**Ectopia**	44 ± 13	0.70 ± 0.51	70 ± 13	13.4 ± 1.3	41
**Total**					75

Anova: **p = 0.002.

Data are expressed as mean ± standard deviation.

### Assessment of visual-motor development

The Bender test shows that children obtained scores that are equivalent to a delay in visual-motor maturation of: AT −3.2 and EC −2.3 years at 9 years of age, significantly different compared to the delay at 8 years of age: AT −2.1 (*p* < 0.001) and EC −1.8 (*p* < 0.05). Comparison between groups shows significant statistical difference at 9 years of age AT −3.2 y, EC −2.3 y (*p* < 0.007). The best scores were recorded at 9 years of age, for children diagnosed with ectopia and although they indicated a slight increase in the age of visuomotor maturation, the delay with respect to the chronological age remained above −2 years. (Table 2).

**Table 2:** Mean scores of the Bender test, Visual-motor maturation index (VMI) and delay in visual-motor maturity.

	8 years			9 years			
	Bender score	VMI	Delay in years	Bender score	VMI	Delay in years	*N*
**Athyrosis**	9 ± 3	74% ± 15%	-2.1 ± 1.2	10 ± 4	65% ± 12%	-3.2 ± 1.1**	34
**Ectopia**	8 ± 3	78% ± 14%	-1.8 ± 1.1	7 ± 3	74% ± 13%	-2.3 ± 1.2*^,**+**^	41
**Total**							75

Anova: **p = 0.001 athyrosis 8 vs 9 years, *p < 0.05 ectopia 8 vs 9 years, ^**+**^*p* < 0.007 athyrosis vs ectopia at 9 years.

Data are expressed as mean ± standard deviation.

The distribution of errors (calculated based on the percentage with which each type of error contributed to the total Bender test score) indicated that the highest percentage corresponded to failures in the integration of parts of the pattern, representing 40% of errors, followed by shape distortion (~26%), rotation of the pattern (~24%) and replacing of dots with circles or lines (~14%); the other errors accounted for less than 10% of errors. Differences attributable to age were recorded in children with athyrosis for shape distortion decreasing from 25% to 17% (*p* = 0.005) and rotation of the pattern increasing from 15% to 24% (*p* < 0.004). A similar finding was observed for the group diagnosed with ectopia for shape distortion decreasing from 26% at eight years to 14% at nine years (*p* = 0.005). The errors in the integration of parts of the pattern and replacement of points with circles or lines were not significantly different between 8 and 9 years of age (Table 3).

**Table 3:** Percentage of errors most frequently recorded in the reproduction of Bender’s test patterns.

	Athyrosis (34)			Ectopia (41)		
	8 y	9 y	*p*	8 y	9 y	*p*
**Integration of parts of the pattern**	40% ± 16%	38% ± 14%	N.S.	36% ± 12%	39% ± 18%	N.S.
**Shape distortion**	25% ± 9%	17% ± 10%	0.005	26% ± 12%	14% ± 12%	0.005
**Rotation of the pattern**	15% ± 11%	24% ± 11%	0.004	14% ± 12%	19% ± 14%	N.S.
**Replacing dots with circles or lines**.	7% ± 9%	6% ± 9%	N.S.	14% ± 13%	13% ± 13%	N.S.

Anova: No differences between groups.

Data are expressed as mean ± standard deviation. (**N**)

### Assessment of intellectual development

For the verbal, performance, and global scales, children diagnosed with athyrosis had the lowest mean scores (between 89 and 84 points), which were significantly lower (*p* < 0.002), both at ages 8 y and 9 y, than the scores of children with ectopia. These children obtained the highest scores on the performance scale (101 points) at 8 and 9 years of age. Children with athyrosis had scores below the cut-off point of normality (<90 points) according to the Wechsler scale. (Table 4).

**Table 4:** Mean Weschler IQ scores.

	Verbal IQ		Performance IQ		Global IQ		
	8 y	9 y	8 y	9 y	8 y	9 y	*N*
**Athyrosis**	87 ± 11**	84 ± 11**	89 ± 12**	89 ± 15**	87 ± 10**	85 ± 13**	34
**Ectopia**	98 ± 12	96 ± 13	101 ± 11	101 ± 14	99 ± 11	98 ± 13	41
**Total**							75

Anova: **p < 0.002 athyrosis vs ectopia

No differences by age. Data are expressed as mean ± standard deviation.

Children with ectopia had a higher cognitive development index than did children with athyrosis both at 8 years of age and at 9 years of age. The intellectual development for athyrosis group represented a delay of −1.3 years in comparison with their chronological age at 9 years of age. The difference between the group with athyrosis and ectopia at 8 and 9 years of age is significantly different (*p* < 0.002) (Table 5).

**Table 5:** Mean Cognitive Development Index and equivalent delay in years.

	8 years		9 years		
	Cognitive development index	Delay in years	Cognitive development index	Delay in years	*N*
**Athyrosis**	89% ± 12%	−0.8 ± 0.9**	85% ± 13%	−1.3 ± 1.2**	34
**Ectopia**	101% ± 11%	1 ± 0.9	99% ± 14%	−0.1 ± 1.2	41
**Total**					75

Anova: **p < 0.002 athyrosis vs ectopia.

No differences by age. Data are expressed as mean ± standard deviation.

### Interaction between visual-motor development and IQ

To evaluate the relationship between Bender test scores and Wechsler test scores, the global IQ of children with CH who did or did not present errors in the reproduction of patterns was compared. In general, children who did not make mistakes had higher IQ scores (*p* < 0.05) than did children who made mistakes for the following errors: pattern distortion cards A, and 7b; failure to integrate all parts of a pattern cards A and 4, drawing perseveration card 6, and pattern rotation cards 5 and 8. Children who did not present errors in the reproduction pattern had higher IQ scores than did children who made errors, being more evident for the ectopia group at 9 years of age (Table 6).

**Table 6:** Global IQ scores according to the absence (0) or presence (1) of errors in reproducing the patterns in the Bender test.

Card	Error		Athyrosis	*N*	Ectopia	*N*
**A**	Pattern distortion	0	87 ± 13	24	100 ± 11	32
		1	78 ± 12	10	90 ± 10*	9
**A**	Pattern integration	0	86 ± 13	26	99 ± 11	36
		1	78 ± 11	8	89 ± 8*	5
**4**	Pattern integration	0	93 ± 14	9	102 ± 10	21
		1	81 ± 11*	25	93 ± 11*	20
**5**	Pattern rotation	0	88 ± 13	20	100 ± 11	27
		1	79 ± 11*	14	93 ± 11	14
**6**	Drawing Perseveration	0	86 ± 12	31	98 ± 12	39
		1	67 ± 3*	3	92 ± 1	2
**7b**	Pattern distortion	0	94 ± 14	10	101 ± 12	20
		1	80 ± 10**	24	94 ± 10*	21
**8**	Pattern rotation	0	98 ± 12	6	104 ± 11	12
		1	81 ± 11**	28	95 ± 11*	29

Anova: *p < 0.05, **p < 0.002.

No differences by age. Data are expressed as mean ± standard deviation.

The correlation analysis of Bender test scores and Wechsler test scores indicated that a higher rate of visual-motor maturation was correlated with IQ. At both ages, there is a positive correlation (p < 0.05) between the visual-motor maturation index and the IQ score. At 8 years of age, the maturation index correlation was: For verbal IQ AT 0.46, EC 0.17; for performance IQ AT 0.42, EC 0.50: and for global IQ AT 0.47, EC 0.39.

At 9 years of age there is an important increase in the maturation index and the correlation was: AT 0.60, EC 0.43 for verbal IQ, AT 0.70; EC 0.58 for performance IQ, and AT 0.68 EC 0.56 for global IQ score.

**Fig. 1:**
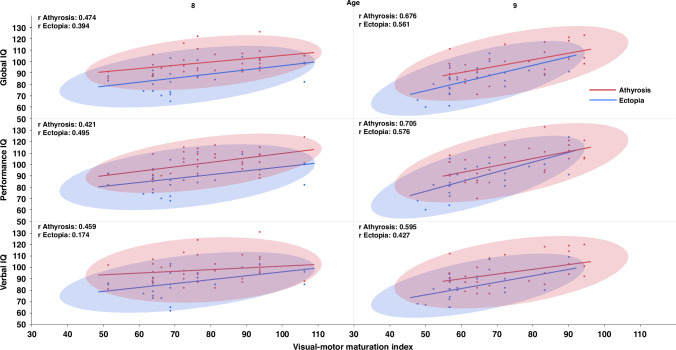
Correlation between the visuo-motor maturation index and the IQ score at 8 and 9 years of age. There is a positive correlation, especially at age 9, for the performance scale in the athyrosis group, for which the r value increased from 0.42 at age 8 to 0.70 at age 9.

In both ages the highest correlation index was registered between visual-motor maturation index and performance IQ. (Fig. 1).

### Effect of the initial L-T_4_ dose on visual-motor and intellectual development

Table 7 shows the visual-motor maturation index, the cognitive development index, and the verbal, performance and global IQ scores as a function of the initial dose of L-T_4_.

**Table 7:** Mean Visual-motor maturation index (VMI), cognitive development index (CDI), and Verbal, Performance, and Global IQ scores by the initial dose of L-T_4_.

	L-T_4_ Dose	VMI	CDI	VIQ	PIQ	GIQ	*N*
**Athyrosis**	High	68% ± 13%	87% ± 13%	85 ± 11	88 ± 13	85 ± 12	30
	Low	75% ± 23%	92% ± 14%	87 ± 13	95 ± 14	90 ± 13	4
**Ectopia**	High	77% ± 14%**	101% ± 12%***	98 ± 13***	103 ± 12***	100 ± 11***	30
	Low	74% ± 12%	95% ± 10%*	94 ± 11*	97 ± 12*	94 ± 11*	11
**Total**							75

Anova: *p < 0.02,**p < 0.001, ***p < 0.0001 ectopia high or low vs athyrosis high.

L-T_4_ Dose: High >12.5 µg/kg/day, Low <12.5 µg/kg/day.

No differences within groups. Data are expressed as mean ± standard deviation.

The analysis of the visual-motor maturation index indicated that children with ectopia who received high L-T_4_ doses had a significantly (*p* < 0.001) higher index (77%) than those who received high doses in the athyrosis group, with a rate of 68%, which is equivalent to a delay of ~3 years with respect to their chronological age; the visual-motor maturation index for the other groups is equivalent to a delay of around −2 years regardless of the L-T_4_ dose administered.

In terms of cognitive development, children with ectopia who received high or low L-T_4_ doses had a significantly higher developmental index (*p* < 0.0001 or *p* < 0.02) than children with athyrosis treated with a high dose of L-T_4,_ who had the lowest value of the developmental index (87%); children with athyrosis treated with low doses of L-T_4_ reach a cognitive development index of 92%, 5 percentage points higher than children with athyrosis and high doses of L-T_4_; however, this difference was not significant. Nevertheless, the delay in cognitive development is less than that recorded in visuomotor development.

Verbal, performance, and global IQ scores were also associated with the initial dose of L-T_4_ administered. Higher verbal, performance, and global IQ scores were observed in children with ectopia treated with high or low doses of L-T_4_; these scores were significantly different (*p* < 0.0001 or *p* < 0.02) from those of children with athyrosis treated with high doses of L-T_4_.

It should be noted that in the athyrotic group, children receiving low doses of L-T_4_ had better IQ than those treated with high doses particularly on the Performance IQ, where the difference reached 7 points. Although the group of children with athyrosis and high doses of L-T_4_ had average scores below 90 points on all scales, the scores did not classify these children as deficient (<75 points on the Weschler scale).

### Evaluation of the effect of the age of treatment initiation and attendance to the development follow-up and intervention program on visual-motor maturation and intellectual development in children with CH

Early initiation of treatment was associated with better visual-motor maturation, while late initiation in the athyrosis group was associated with a lower visual-motor maturation index, corresponding to an age delay of about -3.5 years; especially in this group, the difference was statistically significant (*p* < 0.003) from those with athyrosis and early initiation of treatment, and ectopia independent of the age of initiation of treatment. No significant differences were found in the children diagnosed with ectopia. However, the delay in visual-motor maturation remains at about -2 years.

Cognitive development index was significantly lower (*p* < 0.001), in infants diagnosed with athyrosis with late treatment compared to the other groups. There was a 16 points difference between the athyrosis group of children who started treatment <30 days of age compared to children who started treatment >30 days of age. In the group of children with ectopia, the development rate is even higher than 100%, which means that their intellectual development is in line with their chronological age. Children with ectopia who were treated late showed a 7-points difference compared to children who started treatment <30 days of age.

Analysis of IQ scores showed that children with athyrosis and early treatment initiation had higher VIQ, PIQ, and GIQ scores than late initiators by up to 13 points on the Global IQ; however, the difference was not statistically significant. The difference increased to 17 points on the VIQ, 23 points on the PIQ, and 21 points on the GIQ compared to children with ectopia and early treatment initiation. These differences were statistically significant *p* < 0.003. In the groups with ectopia, the differences were smaller; however, the early initiation group had higher scores, with a difference of 2 points on the verbal scale, 7 points on the performance scale, and 5 points on the global scale. Table 8.

**Table 8:** Visual-motor maturation index (VMI), cognitive development index (CDI), verbal, performance, and global IQ scores by age at the start of the treatment.

	Age at start of treatment	VMI	CDI	VIQ	PIQ	GIQ	*N*
**Athyrosis**	Early	74% ± 13%	96% ± 12%	91 ± 11	97 ± 16	93 ± 13	23
	Late	61% ± 9%***	80% ± 8%***	80 ± 9***	83 ± 11***	80 ± 10***	11
**Ectopia**	Early	80% ± 14%	104% ± 14%	97 ± 12	106 ± 13	101 ± 12	16
	Late	72% ± 12%	97% ± 11%	95 ± 12	99 ± 13	96 ± 11	25
**Total**							75

Anova: ***p < 0.003 athyrosis late vs all groups.

Age at start of treatment: Early <30 days, Late>30 days.

Data are expressed as mean ± standard deviation.

Attendance at the Developmental Follow-up and Intervention Program had a relative effect on the visual-motor maturation of the children in the ectopia group. In this group of children, a higher visual-motor maturation index was recorded (~75%), this difference was statistically significant (*p* = 0.01) from those with athyrosis (64–68%) corresponding to an age delay of about −3.5 years, regardless of the percentage of attendance. No significant differences were found in the children diagnosed with ectopia. However, the delay in visual-motor maturation remained at about −2 years.

Cognitive development indices were significantly higher in children with ectopia with a difference of up than 17 percentage points (*p* < 0.001), compared with infants diagnosed with athyrosis regardless the percentage of attendance; the indices of cognitive development in this last group of children (~86%) are equivalent to a delay of approximately -1 year. Once again in the group of children with ectopia, the development rate is even higher than 100%, which means that their intellectual development is in line with their chronological age. ;IQ scores showed that children with athyrosis and >80% attendance had higher VIQ, PIQ, and GIQ scores than children with athyrosis and <80% attendance by up to 8 points on the Performance IQ; however, the difference was not statistically significant. The difference between the children with athyrosis and the children with ectopia was significant *p* < 0.05 regardless of the percentage of attendance on the VIQ, PIQ, and GIQ. In the groups with ectopia, the differences were smaller; however, the group with >80% attendance had higher scores, with a difference of 4 points on the verbal scale and 5 points on the performance or global scale. Table 9.

**Table 9:** Visual-motor maturation index (VMI), Cognitive development index (CDI), Verbal, Performance and Global IQ by attendance at development monitoring and intervention program appointments.

	Attendance	VMI	CDI	VIQ	PIQ	GIQ	*N*
**Athyrosis**	**>80%**	64% ± 11%	86% ± 12%	85 ± 12	90 ± 12	86 ± 12	23
	**<80%**	68% ± 14%	83% ± 13%	81 ± 10	82 ± 17	80 ± 14	11
**Ectopia**	**>80%**	75% ± 14%*	100% ± 12%**	98 ± 13**	104 ± 13**	101 ± 12**	16
	**<80%**	74% ± 13%*	98% ± 12%**	94 ± 11**	99 ± 13^++,+^	96 ± 11^++,+^	25
**Total**							75

Anova: *p < 0.01, **p < 0.001 vs athyrosis ><80%. ^++^*p* < 0.001 vs athyrosis <80%, ^+^*p* < 0.05 vs athyrosis >80%.

No differences within groups. Data are expressed as mean ± standard deviation.

## Discussion

### Visual-motor and intellectual development in children with CH

According to Urzúa,^47^ maturity is defined as “the level of organization and development that allows the development of cognitive and behavioral functions according to the chronological age of the subject.” Rincón^48^ points out that “the maturation process that occurs during childhood is related to the process of brain development, hence the importance of neurological and neuropsychological maturation as directly responsible for the cognitive efficacy of school-age children”. Graphical-constructive tests are characterized by requiring a fine and precise skill that is consolidated from repeated practice^45^; therefore, herein, the application of the Bender test was highly relevant considering that it provided important information about visual-motor development in this population.

The normative data of the Bender test indicate that the expected score for children between 8 and 9 years of age range from 2 to 4 errors in the reproduction of patterns.^49^ Our results showed that the scores for children with CH ranged from 7–10 errors, indicating that these children performed poorly on this test. Increased scores resulted from committing a greater number of errors, which translates into a delay in visual-motor maturation. The visual-motor development indices showed that children with CH did not reach the visual-motor maturity corresponding to their chronological age and were below 80% of that expected for age. These results are consistent with those of studies in which visual-motor development was evaluated. Such studies reported that children with CH presented lower performance in information processing, reaction time, visual-construction and execution of motor skills; the results support the notion that CH affects the neuropsychological domains that involve these functions and that the damage persists despite the implementation of appropriate therapeutic strategies.^19–21^

Visual-motor integration and control involves the activation of brain regions such as the posterior parietal cortex, the cerebellum, and the corpus callosum. These regions work together to activate or deactivate internal motor models for the lateralization of visual-motor functions and for the interhemispheric communication of visual-motor information.^50^ The data obtained in our study show the existence of a significant delay in the visual-motor maturity of school-age children with CH. Although the precise causes of the deficit in these patients are unknown, clinical studies suggest that CH induces alterations in the neural substrates involved in visual-motor integration. In this regard, thickening has been evidenced in various cortical gyri, including the superior parietal gyrus, in children with CH.^16^ At a functional level, CH causes a decrease in intracortical conduction speed,^51,52^ reduces the activation of the inferior parietal cortex and modifies the activation pattern when children perform a mental rotation task.^22^ These brain alterations could partially explain the visual motor deficit in children with CH because the deficiency of thyroid hormones during the perinatal period could lead to brain morphofunctional changes that remain despite treatment with L-T_4_.

The deficit in the visual-motor domain in children with CH is relevant because this domain influences cognitive functions. Particularly in the Bender test, the quality of the reproduction of geometric patterns is closely related to the development of skills such as reading and writing. Most of the errors identified were those related to the failure to integration all parts of the pattern or distortion of the shape of the pattern. In this regard, Fuentes^53^ reported that the errors made in the execution of strokes, lines, or geometric patterns are decisive for reading-writing processes and that the presence of alterations in these activities can predict problems in written language or reading, which will inevitably be reflected in the development of cognitive skills related to learning processes. In the same sense, the study carried out by Simic^34^ showed that individuals with CH, diagnosed in a timely manner and with early initiation of treatment, had lower scores than did their peers by age and sex in direct tests of visual-cognitive function, such as judging the orientation of a line, the location of parts in the whole and copying towers of three-dimensional blocks; additionally, the presence of these alterations was reflected in psychomotor immaturity. In addition, when applying the Bender test in children 4–6 years of age, Zambrano^54^ reported that children of low socioeconomic status between 4 and 6 years of age had a mean visual-motor age lower by 1 year with respect to chronological age and that this delay was identified as an important risk factor for the development of learning disabilities.

Regarding the evaluation of intellectual development, the results revealed that the IQ mean scores of children with ectopia were within the limits of normal development (90–109 points, Wechsler scale), but not for children with athyrosis, whose IQ mean scores were below 90 points. IQ is one of the most studied variables in children with CH, and our data are consistent with the results of previous studies, i.e.; the average verbal, performance, and global IQ scores are significantly lower when treatment is started after 15 days postnatal.^25^ It should be noted that the results of this study demonstrated that even if treatment started at 30 days of age, but not later, there is acceptable cognitive development since the mean scores did not classify these children as deficient (<75 points according to the Weschler scale).

Koppitz^45^ reported the existence of a significant correlation between Bender test and Wechsler Intelligence test performance, which is why the Bender test has been considered a brief instrument for the assessment of nonverbal intelligence. In our study, the analysis of the correlation ratios between the type of errors identified by the Bender test and the score of the verbal performance and global intelligence test scales allowed the identification of the presence of visual-motor developmental alterations is significantly associated with intellectual development, particularly with the performance scale. The above results reveal that errors in the reproduction of the patterns may be indicative of alterations in intellectual development. In this sense, Cruz^55^ suggested that the measurement of visual-motor ability at school age can contribute to the approximate prediction of intelligence, considering that age impacts both domains.^38^

In the bibliographic review, there were no specific results reported for children with CH treated with L-T_4_ (early or late) using a specific test such as the Bender-Gestalt test or Koppitz scoring system to determine the level of maturity of visual-motor development in the school stage. This test provided interesting data on the most frequent visual-motor errors and the visual-motor maturation age of children with CH.

### Children with Athyrosis: a group with more severe sequelae

Children with athyrosis and delayed treatment have more severe sequelae in both visual-motor and intellectual development. Visual-motor development is significantly delayed in these patients; their visual-motor maturation rate was 65% at 9 years of age, corresponding to a delay of -3.2 years. Children with athyrosis had significantly lower scores (between 84 and 89 points) in verbal, performance and global scales than those for ectopia group. This fact suggests that cognitive development in children with athyrosis is closely related to the severity of CH and the initiation of hormone substitutive treatment. This is a common finding in studies that have evaluated the sequelae of CH on intellectual development; such studies have reported that patients with CH due to athyrosis have greater IQ deficits than do children with CH due to ectopy^8,56,57^ and that IQ is reduced in children with CH who receive late treatment.^25^ Our findings support that a child with athyrosis and late treatment is at risk of developing severe cognitive and motor impairment and an IQ at least one standard deviation below the reference mean,^44^ as has already been reported previously by our group and other researchers.^7,58^

### Initial L-T_4_ dose effect on visual-motor and intellectual development

Our results showed that children diagnosed with athyrosis who received L-T_4_ doses >12.5 µg/kg/day had the greatest delay in visual-motor and developmental maturation and the lowest IQ scores. These results are consistent with previous findings showing that severe CH is associated with greater deficits in visuospatial and visuoconstructive skills^29^ and that the initial overdose of L-T_4_ (at doses greater than 10 µg/kg/day) results in a decrease in IQ scores in children aged 2 to 11 years.^59,60^ Although it is true that the current recommendation for children with severe CH is an initial L-T_4_ dose of 10–15 µg/kg/day,^1^ our results suggest that doses greater than 12.5 µg/kg/day may be inadequate in such patients and may interfere with visual-motor and developmental maturation. In contrast, the administration of lower doses of L-T_4_ has a better effect on visuomotor maturation and few adverse effects on IQ, perhaps because the probability of overdose events is reduced. In a recent study, He et al. ^61^ suggested that an individualized dose of levothyroxine can provide the same therapeutic effect as the recommended dose and that this strategy can also reduce the risk of an overdose.

Importantly, children with severe CH who received doses of L-T_4_ less than 12.5 µg/kg/day showed better performance on the test of intellectual development. In this regard, Günbey et al. ^62^ reported that some patients with moderate and severe CH experienced iatrogenic hyperthyroxinemia; however, the dose was near the lower limit of the recommended range, thus suggesting that lower initial doses may be appropriate under strict follow-up.

### Importance of the attendance development follow-up and intervention program

The data show that program attendance has a relative effect on visual-motor maturation in the different subgroups of patients with CH; although better indices were observed in children who attended more than 80% of the appointments, visual-motor maturation delay remains important.

Regular attendance to the program reduces the difference between developmental age and chronological age resulting in chronological age development in children who attended more than 80% of the scheduled appointments.

The results also showed a relatively beneficial effect of the developmental follow-up and intervention program in children who attended more than 80% of the scheduled appointments. For example, in the group of children with athyrosis, even though the differences are not significant, IQ was better in the group of children who regularly attended the intervention program appointments. Evidence suggests that programs that promote high-quality parent-child interactions and provide cognitively and linguistically enriched learning experiences have a positive impact on young children’s cognitive development and academic achievement.^40–42, 63^

Sociocultural elements such as stimulation at home, socioeconomic status, and education of parents or caregivers^64^ play an important role in the intellectual development of children, especially those suffering from CH. The patients included in this study came from families whose socio-cultural and economic levels were classified as medium-low or low according to the scale used by the National Institute of Pediatrics; therefore, the children in the study were considered a vulnerable population. In this sense, our data support the idea that all children with athyrosis who start treatment late should be included in a neurodevelopmental follow-up program and receive neuropsychological interventions, given the importance of the developmental functions involved. The specialized care they receive and, more importantly, the active participation of parents in the development of their own children may act as a favorable factor for cognitive development and mitigate learning problems.^58^

## Conclusions

Children with CH and late onset of treatment show a delay in visual-motor maturation. This is associated with a reduction in IQ. Timely diagnosis and early treatment with the appropriate dose of L-T_4_ are critical factors in limiting the sequelae of CH; inclusion in a developmental follow-up and intervention program that promotes cognitive and visual-motor development is highly recommended, especially for those patients with the severe form of CH. Finally, the results suggest that the assessment of visual-motor development may be helpful in detecting alterations that will later be reflected in the academic performance of patients with CH.

## Study limitations

The international recommendation establishes treatment initiation at 15 days of life; however, we arbitrarily established a cut-off point of 30 days to evaluate the effect of treatment initiation, so there was no opportunity to compare their evolution with that of patients treated early; therefore, the results of our study lack this important comparison group.

The Developmental Follow-up and Intervention Program is designed to provide neurodevelopmental follow-up of children from the time of diagnosis until 14 years of age; however, we only included follow-up data from 8 to 9 years of age, which obviously limits the conclusions about the benefits of follow-up and early stimulation.

The patients’ families were in the low and lower-middle socioeconomic levels, so the impact of the full range of socioeconomic variables on cognitive development, particularly language development, may not be fully reflected.

## Data Availability

The data analyzed in this study is suitable upon reasonable request. Requests to access these datasets should be directed to Gerardo Barragán. mbarraganm@pediatria.gob.mx
